# Association between blood groups and myocardial injury after non-cardiac surgery: a retrospective cohort study

**DOI:** 10.1038/s41598-024-61546-w

**Published:** 2024-06-18

**Authors:** Jinze Li, Wangyu Li, Longyun Li, Shengze Yang, Guoqing Zhao, Kai Li

**Affiliations:** 1 Department of Anesthesiology, China-Japan Union Hospital, Jilin University, 126th Xiantai Avenue, Changchun, 130033 China; 2 Department of Anesthesiology, Beijing Anzhen Hospital, Capital Medical University, Beijing, China; 3 Department of Pain Management, Fujian Provincial Hospital, Shengli Clinical Medical College of Fujian Medical University, Fuzhou, China; 4 Jilin University, Changchun, China; 5 College of Anesthesiology, Xuzhou Medical University, Jiangsu Provincial Key Laboratory of Anesthesia and Analgesia, Xuzhou, China

**Keywords:** Myocardial injury after non-cardiac surgery (MINS), Blood type, Path analysis, Cardiovascular risk, Cardiology, Medical research, Risk factors

## Abstract

Blood group is a potential genetic element in coronary artery disease. Nevertheless, the relationship between different ABO blood groups and myocardial injury after non-cardiac surgery (MINS) is poorly understood. This study verified whether ABO blood group is a potential MINS influencing factor. This retrospective cohort study included 1201 patients who underwent elective non-cardiac surgery and a mandatory troponin test on postoperative days 1 and 2 from 2019 to 2020 at a university-affiliated tertiary hospital. The primary outcome was associations between ABO blood groups and MINS, assessed using univariate and multivariate logistic-regression analyses. Path analysis was used to investigate direct and indirect effects between blood group and MINS. MINS incidence (102/1201, 8.5%) was higher in blood-type B patients than in non-B patients [blood-type B: 44/400 (11.0%) vs. non-B: 58/801 (7.2%); adjusted odds ratio = 1.57 (1.03–2.38); *p* = 0.036]. In the confounding factor model, preoperative hypertension and coronary artery disease medical history were associated with MINS risk [adjusted odds ratio: 2.00 (1.30–3.06), *p* = 0.002; 2.81 (1.71–4.61), *p* < 0.001, respectively]. Path analysis did not uncover any mediating role for hypertension, diabetes, or coronary artery disease between blood type and MINS. Therefore, blood-type B is associated with higher MINS risk; potential mediators of this association need to be investigated.

## Introduction

Oligosaccharide structures determine ABO blood groups, and the composition of oligosaccharides is controlled by the genes encoding glycosyltransferases. Blood group antigens have different phenotypes and gene-derived glycoconjugate structures, and these glycoconjugate structures play a crucial role in the physiopathology of cells^1,2^. In the past, ABO antigens were believed to be only present on the outer surface of erythrocytes. However, ABO antigens have been shown to be expressed not only by erythrocytes but also by various human tissues and cells such as epithelial cells and vascular endothelial cells^3,4^. Thus, the ABO blood group plays a crucial role in various diseases, and previous studies have demonstrated that it is not only a risk factor for atherogenesis, but also an important factor in the pathogenesis of acute coronary syndrome and myocardial infarction^5,6^. The hypothesized mechanism may involve the influence of individual susceptibility to disease by modulating the hemostatic system and inflammatory response^7^. Myocardial injury after non-cardiac surgery (MINS) appears to result from myocardial supply–demand mismatch and is mainly confined to patients with a pre-existing risk of coronary artery disease^8^. The diagnostic criterion is a troponin (cTn) concentration above the upper 99th percentile reference limit for cTn measurements during or within 30 days after non-cardiac surgery, although this almost always occurs within the first 2 days after surgery. In most patients with myocardial injury, troponin elevations are not accompanied by symptoms, but are associated with a significant increase in mortality 30 days after surgery^9,10^. According to the latest guidelines for MINS, risk factors for perioperative MINS include diabetes, coronary artery disease, and hypertension. Additionally, previous studies have linked ABO blood groups with risk factors for MINS; however, the O blood group was primarily compared with the non-O blood groups, and cardiovascular risk was higher in the non-O blood groups^11,12^. Among the non-O blood groups, the cardiovascular risk for blood type B is higher than that for other blood types^13–15^. The association between ABO blood type and MINS has not yet received widespread attention. Thus, based on these initial results, we hypothesize that the risk of MINS is higher in patients with blood type B compared with those with other blood types.

## Results

### Patients

**Figure 1:**
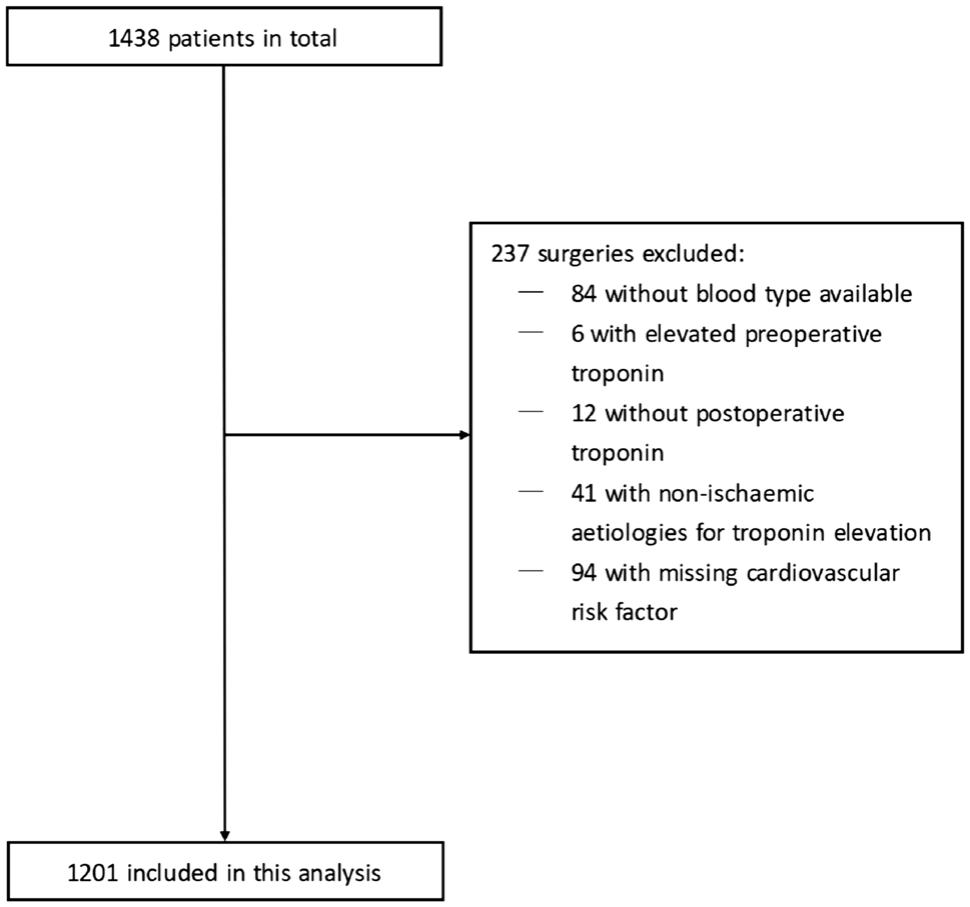
Patient selection flowchart.

We screened 1438 patients who underwent non-cardiac surgery at CJUH between 2019 and 2020, of which 84 patients were excluded due to missing blood group data, another 6 patients for elevated preoperative troponin, 12 patients for missing postoperative troponin, 41patients for non-ischaemic aetiologies for troponin elevation, and additionally 94 patients were excluded due to missing cardiovascular risk factor, and finally a total of 1201(83.5%) patients were included in the analysis (Fig. 1). The baseline characteristics of the cohort are presented in (Table 1; mean age: 64.9 (8.8) years, male: 60.6%).

**Table 1:** Patient demographic, medical, and surgical characteristics according to the occurrence of MINS.

Variables	Whole cohort (n = 1201)	O (n = 351)	A (n = 336)	B (n = 400)	AB (n = 114)
Age (yr), mean (SD)	64.9 (8.8)	64.8 (8.8)	64.7 (8.7)	65.2 (9.0)	64.6 (8.7)
Sex (male, %)	728 (60.6)	221 (63.0)	205 (60.8)	234 (58.5)	69 (60.5)
BMI (kg m^−2^), mean (SD)	23.7 (3.5)	23.7 (3.3)	23.9 (3.4)	23.7 (3.6)	23.4 (3.6)
Smoking (%)	512 (42.6)	147 (41.9)	156 (46.4)	165 (41.0)	45 (39.5)
Comorbidities (%)					
Coronary artery disease	145 (12.1)	39 (11.1)	44 (13.1)	50 (12.5)	12 (10.5)
Hypertension	483 (40.2)	136 (38.7)	132 (39.3)	162 (40.5)	53 (46.5)
Diabetes mellitus	212 (17.7)	59 (16.8)	61 (18.2)	74 (18.5)	18 (15.8)
Cerebrovascular diseases	147 (12.2)	49 (14.0)	40 (11.9)	43 (10.8)	15 (13.2)
Surgical procedure type (%)					
Hepatobiliary	69 (5.7)	19 (5.4)	19 (5.6)	23 (5.7)	8 (7.0)
Urinary	92 (7.6)	24 (6.8)	28 (8.3)	31 (7.7)	9 (7.9)
Colorectal	361 (29.9)	111 (31.6)	104 (30.1)	108 (26.9)	38 (33.3)
Spine	124 (10.2)	29 (8.2)	41 (12.2)	41 (10.2)	13 (11.4)
Thoracic	193 (16.0)	48 (13.6)	59 (17.5)	70 (17.4)	16 (14.1)
Preoperative serum creatinine (μmol L^−1^), median (IQR)	65.1 (55.8; 75.8)	65.9 (55.6; 76.4)	64.5 (55.9; 76.3)	64.7 (55.4; 75.2)	65.4 (56.4; 75.4)
Medication use (%)					
Beta blockers	27 (2.2)	9 (2.6)	5 (1.5)	12 (3.0)	1 (0.9)
ACE inhibitors	10 (0.8)	4 (1.1)	1 (0.3)	3 (0.8)	2 (1.8)
Aspirin	15 (1.2)	4 (1.1)	6 (1.8)	4 (1.0)	1 (0.9)
Insulin	63 (5.2)	18 (5.1)	23 (6.8)	19 (4.8)	3 (2.6)
CCB	128 (10.7)	31 (8.8)	40 (11.9)	47 (11.8)	10 (8.8)
Blood loss (ml), median (IQR)	200 (100; 300)	200 (100; 400)	200 (100; 400)	200 (100; 300)	200 (100; 300)
Preoperative hemoglobin (g L^−1^), median (IQR)	137 (123; 149)	140 (124; 150)	136 (123; 148)	137 (124; 149)	135 (118; 150)
Preoperative WBC	6.3 (5.2; 7.6)	6.3 (5.4; 7.6)	6.3 (5.2; 7.6)	6.2 (5.1; 7.7)	6.4 (5.1; 7.7)

Statistics are expressed as count (percentage), median (inter-quartile range), or mean (standard deviation). ACE, angiotensin-converting enzyme; WBC, white blood cells; IOR, interquartile range; CCB, calcium channel blocker; MINS, myocardial injury after non-cardiac surgery.

### Incidence and risk factors of MINS

The number of patients with MINS was 102/1201 (8.5%), and after screening all baseline and perioperative variables by univariate analysis, we included variables that were analyzed univariately at *P* < 0.05 or were considered clinically significant in the multivariate logistic regression model. After adjusting for hypertension, diabetes, coronary artery disease, BMI, cerebrovascular disease, and sex, the rate of MINS in patients with blood type B was 44/400 (11.0%) vs. non-B blood type: 58/801 (7.2%); 1.57 (1.03–2.38); *p* = 0.036 (Table 2). In the risk factor model associated with MINS, hypertension and coronary artery disease were independently associated with the risk of MINS [OR 2.00 (1.30–3.06), *p* = 0.002; 2.81 (1.71–4.61), *p* < 0.001] (Supplementary Table 1). In models with different MINS risk factors, patients with blood type B had a numerically higher risk of developing MINS-related risk factors; however, the difference was not statistically significant (Supplementary Fig. 1 and Supplementary Table 2).

**Table 2:** Multi-factor logistic regression analysis of blood group and MINS, adjusted for MINS risk factors.

Blood type	Univariable		*p*	Multivariable		*p*
	OR	(95% CI)		OR	(95% CI)	
O	1.00					
A	1.01	(0.58–1.76)	0.982	0.94	(0.53–1.67)	0.841
B	1.48	(0.90–2.45)	0.124	1.41	(0.85–2.35)	0.189
AB	0.55	(0.21–1.47)	0.232	0.5	(0.19–1.35)	0.173
Non-B	1.00					
B	1.58	(1.05–2.39)	0.029	1.57	(1.03–2.38)	0.036

### Path analysis

**Figure 2:**
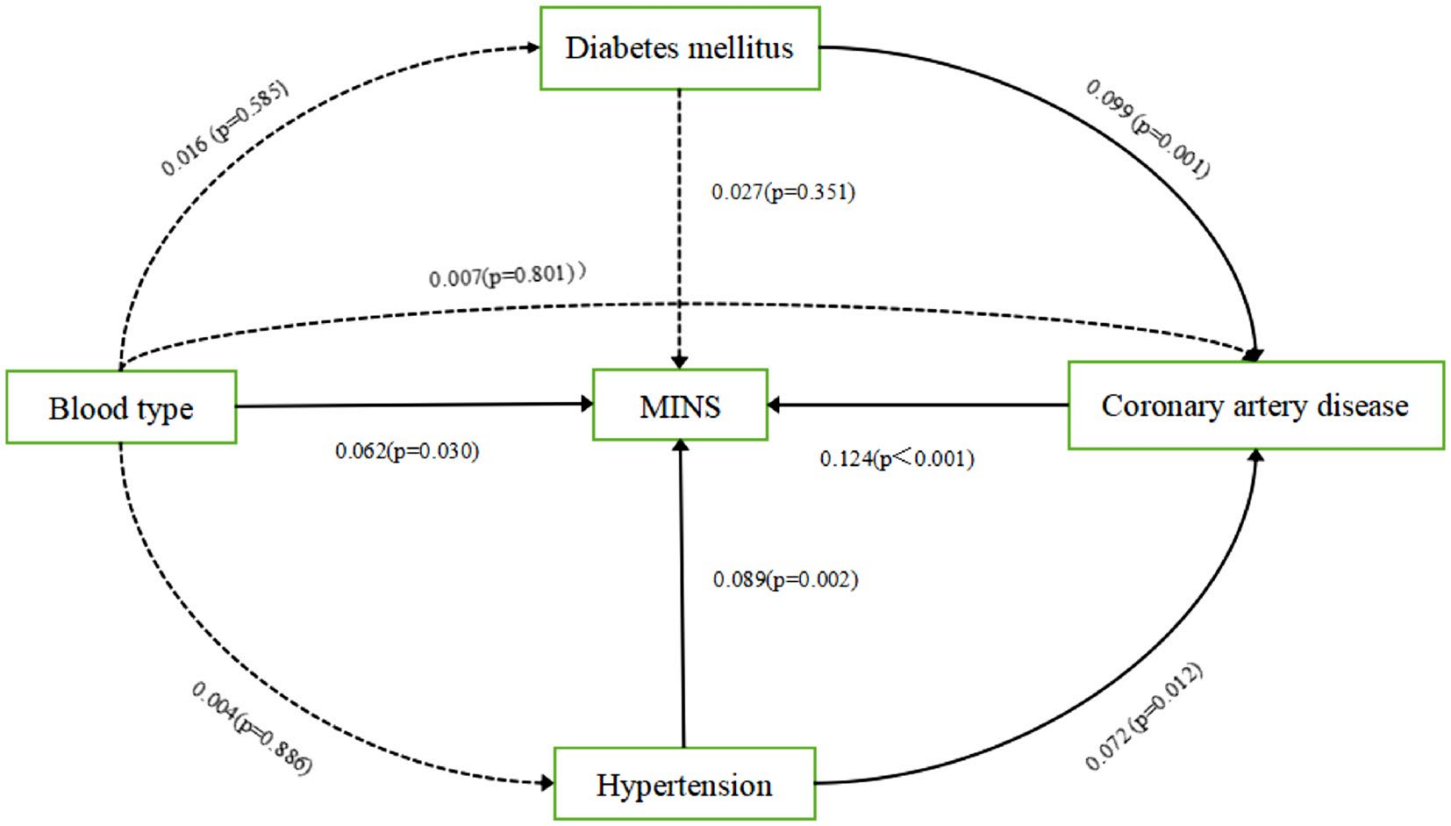
Structural equation modeling to illustrate the relationship between MINS and blood type, diabetes, hypertension, and coronary artery disease.

The magnitude of the path coefficient in the path analysis model indicates the relationship between the variable and the degree of influence of the dependent variable. Positive and negative values represent the positive and negative effects of the influence trend, respectively. Blood type was found to be statistically significant only in the path of MINS at *p* < 0.05, while the other paths were not statistically significant, indicating that diabetes, coronary artery disease, and hypertension did not have a mediating effect on the causal effect of blood type and MINS in the structural equation model, and that the total effect of blood type and MINS was equal to the direct effect (Fig. 2).

### Subgroup analysis

**Figure 3:**
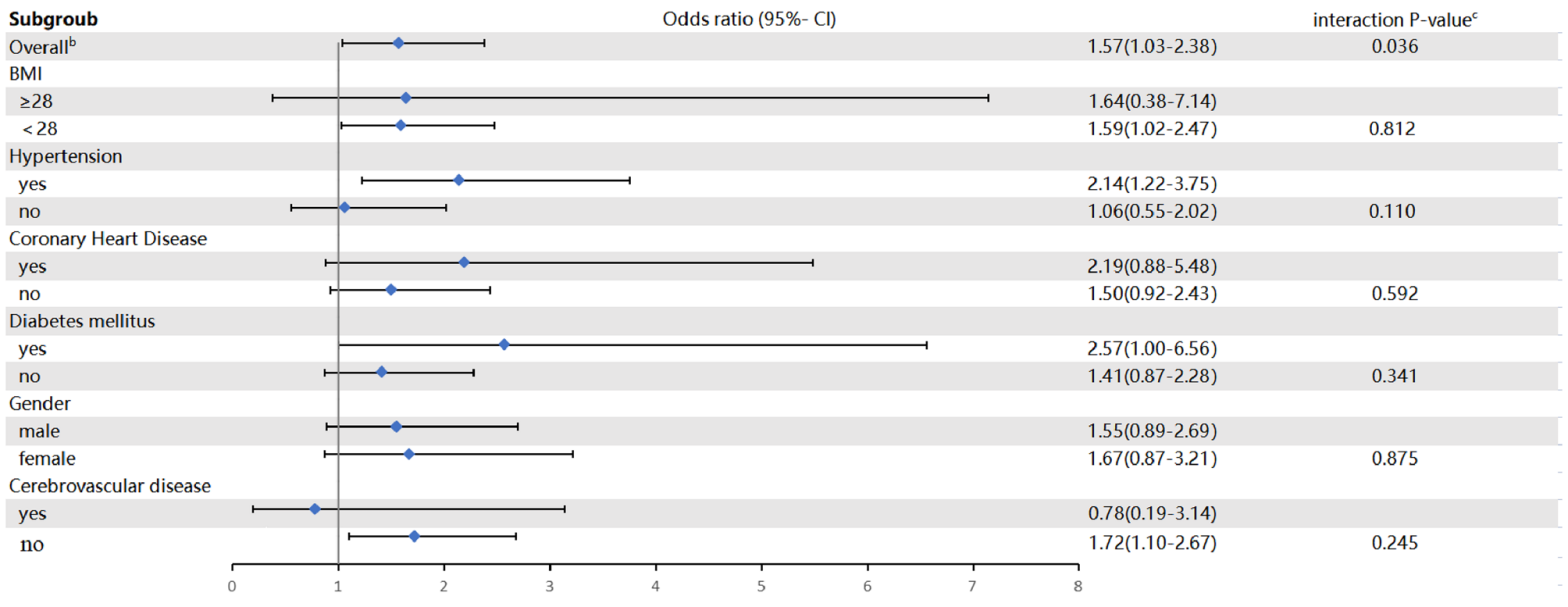
Forest plots of predefined subgroups to estimate the relationship between blood group^a^ and MINS. ^a^Differences in risk of MINS between B and non-B blood types. ^b^All included patients (1201 individuals). ^c^Blood type-by-subgroup interaction.

None of the interaction terms in the predefined subgroups were statistically significant (Fig. 3).

## Discussion

ABO blood groups are genetically determined, have a well-defined phenotype, and have been shown to be associated with hypertension, diabetes, and circulatory disease^3,11,14^. Our retrospective analysis demonstrated an independent correlation between blood groups and the risk of MINS, even after adjusting for risk factors, including hypertension, diabetes, and coronary artery disease. Patients with blood type B had a higher risk of developing MINS than those with non-B blood types. Even though this difference is limited, it adds further information to the existing research^15^. Consistent with our results, a prospective cohort study that included 3823 patients found that patients with non-O blood types had a significantly higher risk of cardiovascular events. The risk was highest for blood type B, followed by blood types AB and A, with HRs (95% CI) of 1.481 (1.122–1.955), 1.249 (0.852–1.831), and 1.083 (0.797–1.472), respectively, compared with blood type O^15^. Moreover, we demonstrated a correlation between hypertension and MINS. The physiological basis for chronic hypertension-promoting myocardial injury is well established, with chronic pressure overload leading to left ventricular hypertrophy and coronary vascular remodeling. Furthermore, hypertension can lead to myocardial ischemia, even in patients with very mild or no coronary stenosis^16–18^. Type II diabetes causes oxidative stress through hyperglycemia and mediates myocardial injury via an imbalance in the production and elimination of free radicals^19,20^. However, our study did not find an association between diabetes and MINS, probably because we did not consider preoperative blood glucose concentrations in non-diabetic patients and because MINS is related not only to diabetes itself but also to preoperative blood glucose concentration. A more robust relationship between preoperative blood glucose concentration and MINS exists in non-diabetic patients than in those with diabetes; for people without known diabetes before surgery, a fasting glucose level of > 6.41 mmol/L can help in predicting MINS^21^. Although we assessed the incidence of MINS, the ABO blood group may have contributed to the development of MINS by influencing certain potential risk factors. For example, certain blood types may increase the risk of hypertension, diabetes, and coronary artery disease, which in turn affects the balance of oxygen supply and demand to the myocardium. Therefore, we performed a subgroup analysis to assess whether the effect of blood type on MINS is associated with MINS risk factors; however, none of the interaction terms reached statistical significance. Blood group is the most basic factor influenced by genetics. Therefore, we performed further pathway analysis but failed to find a mediating role of diabetes, hypertension, and coronary artery disease between blood group and MINS; nonetheless, the association of blood group B with hypertension and diabetes has been established previously^11,14^. Our findings have important implications for understanding MINS, as we found that blood group, a genetically determined risk factor, influences the incidence of MINS, suggesting that some patients have blood group-related susceptibility factors for MINS. Most postoperative myocardial injuries are largely due to a mismatch between myocardial oxygen supply and demand or or atheromatous plaque disruption^8^; therefore, blood type may affect the oxygen supply and demand to the myocardium or promot atherosclerotic plaque formation through several underlying pathophysiological mechanisms, causing myocardial injury. For example, von Willebrand factor (VWF) plasma levels and function are higher in patients with blood type B than in those with other blood types^22,23^. VWF plays an important role in hemostasis and thrombosis by mediating platelet adhesion to vessel walls. Together with fibrinogen, VWF is involved in platelet aggregation and plays a role in the development of atherosclerosis, which is associated with a high risk of heart ischemia and thromboembolic disease^24–26^. In addition, blood group may determine the levels of P-selectin, a potential factor in the formation of atherosclerosis^27,28^. Among the non-O blood types, P-selectin levels were the highest in the B blood type^29^. These results suggest a genetic component contributing to the occurrence of myocardial injury in patients.

### Limitations

Our study had several potential limitations. First, the study was retrospective in nature and, therefore, limited by the type and quality of data available in the medical records, which limits causal inference. Although we adjusted for several potential confounders in the association between blood group and outcome, the limited number of patients diagnosed with MINS prevented us from adjusting for all observed confounders and made it difficult to exclude unobserved confounders. However, ABO blood groups are genetically encoded and do not change with clinical intervention, and no current medical interventions except transfusion and donor-recipient transplant matching are guided by ABO blood types. Therefore, differences in preoperative comorbidities may be a causal or direct consequence of the ABO blood type. Furthermore, the random nature of the genetic distribution of the blood group genes allowed the unobserved confounders to follow the same distribution, which helped mitigate the effect of unobserved confounders. Second, we only analyzed troponin values during the first 2 days after surgery. However, we excluded patients with preoperative troponin abnormalities; over 90% of MINS cases occurred within the first 2 days postoperatively, and only 0.6% of MINS cases were diagnosed beyond the third postoperative day^10^. As such, we only analyzed troponin values within the first 2 days after surgery. Different definitions of MINS may lead to clinical heterogeneity, with recent studies reporting a higher rate of diagnosis of MINS when using high-sensitivity troponin T analysis than troponin I analysis^30^. We were unable to fully identify cases of MINS. The true incidence of MINS may have been underestimated in this study, potentially reducing the observable blood group effects in our data. However, our aim was not to determine incidence. The reported relationship between blood group and myocardial injury would not differ from the complete data unless there was a significant bias. Selection bias is an important factor that must be considered. Our study was a single-center trial in China, with geographic and ethnic biases that may account for our failure to find an association between blood type, hypertension, diabetes, and possibly other mediating factors that we did not consider.

## Conclusion

We observed an association between the ABO blood group and MINS, but whether this relationship is causal remains to be determined. Further studies are needed to determine the underlying pathophysiological mechanisms between the blood group and MINS and to ensure that various confounding factors, including racial factors, exist.

## Materials and methods

### Data gathering and patient population

This study was conducted in compliance with the postulates of Declaration of Helsinkiwas and approved by the Research Ethics Committee of the China-Japan Union Hospital of Jilin University (CJUH) (No. 2023072603). We analyzed the electronic medical records of inpatients at the China-Japan Union Hospital and waived the requirement for informed consent. This retrospective cohort study included patients who underwent elective non-cardiac surgery and with mandatory troponin tests on postoperative days one and two between 2019 and 2020. The following criteria were also met for inclusion: age 45 years or older; non-cardiac surgery under general or regional anesthesia; and at least one cardiovascular risk factor, such as hypertension, diabetes mellitus, stroke, or cardiovascular disease. The exclusion criteria were as follows: no documented blood group and elevated preoperative troponin levels. Clinical information, such as age, sex, medical history, family history, blood group, medication use, and smoking status, was collected at the time of patient admission but was backward reviewed as long as patients were included in the retrospective cohort.

### Primary outcome

The primary outcome was MINS, which was defined as at least one serum troponin I (TnI) level above the 99th percentile of the upper reference limit (0.04 ng/L) within 2 days after surgery. However, patients with elevated troponin due to non-ischemic etiologies, such as sepsis, pulmonary embolism, atrial fibrillation, cardioversion, etc., should be excluded^9,31^. ABO blood groups are determined from our blood bank or laboratory using standard clinical methods.

### Statistical analysis

All statistical analyses were performed using SPSS 25.0. Dichotomous variables were expressed as frequencies and percentages, and tests of normal distribution for continuous variables were performed using the Shapiro–Wilk test. Normally distributed data were expressed as means (standard deviation). Non-normally distributed data were expressed as medians (interquartile ranges).

We used univariate logistic regression analysis to measure the unadjusted association between blood group and myocardial injury. Subsequently, after adjusting for smoking, coronary artery disease, hypertension, diabetes mellitus, sex, and cerebrovascular disease, we constructed a multivariate logistic regression model to determine the association between blood type and myocardial injury. We also developed separate models for diabetes, hypertension, and coronary artery disease and used multivariate logistic regression to analyze the relationship between blood group and risk factors associated with MINS. The results of the logistic regression analysis were presented as odds ratio (OR) with a 95% confidence interval, and a two-sided *p* value < 0.05 was regarded as statistically significant.

### Path analysis

Path analysis is a type of structural equation modeling (SEM) that is typically tested through regression analysis^32^. A series of tests and analyses were performed to obtain the most appropriate model to represent the multiple and complex relationships between the independent and other variables. We used pathway analysis to investigate how the causal effect of blood group on MINS was classified as an indirect and direct effect, and hypothesized that blood type, diabetes mellitus, coronary artery disease, and hypertension are risk factors that affect the dependent variables (MINS). As blood type is the most fundamental variable and is not affected by other variables, hypertension and diabetes mellitus are lower-order variables that are affected by blood type, whereas coronary artery disease is a higher-order variable that is affected by the remaining variables. We defined the indirect effect as the change in the expected incidence of MINS when the blood type was fixed, but the risk factors for MINS, such as hypertension, diabetes, and coronary artery disease, changed when the blood type was changed. We defined the direct effect as the change in the expected incidence of MINS when the blood type changed, whereas the risk factors for MINS related to blood type were artificially fixed. We modeled the incidence of MINS using logistic regression models, with blood type and risk factors for MINS as independent variables, and associated these variables with specific coefficients to form a path analysis plot regression equation. The indirect effect coefficient estimated the strength of the blood type-mediated effect on risk factors for MINS (i.e., how much of the blood type-induced increase in MINS can be attributed to blood type-related risk factors for MINS). The direct effect coefficient estimates the strength of the direct effect of blood type on MINS (i.e., any effect of blood type on MINS that is not mediated by the risk factors for MINS). The total effect coefficient was the sum of the direct and indirect relationships between blood type and MINS.

### Subgroup analysis

We performed a subgroup analysis by dividing the patients into subgroups based on body mass index (BMI), history of diabetes, hypertension, coronary artery disease, cerebrovascular disease, and sex, within each subgroup. An interaction test was used to assess the effect of blood type on MINS.

### Supplementary Information

Supplementary Information.

## Data Availability

The datasets analyzed during this study are available from the corresponding author upon reasonable request.
