# Supplementary material for: Association between blood groups and myocardial injury after non-cardiac surgery: a retrospective cohort study
Source: Sci Rep. 2024 Jun 18;14:14028. doi: 10.1038/s41598-024-61546-w (PMC11189574; doi:10.1038/s41598-024-61546-w)
Supplement: Supplementary file 1 — Supplementary Information. [file 41598_2024_61546_MOESM1_ESM.docx]

**Supplementary Figure 1**. Frequency distribution of MINS risk factors in different blood groups

.
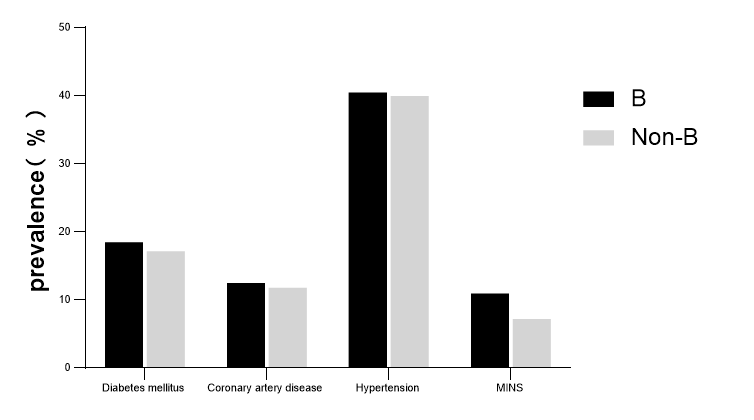


**Supplementary Table 1.** Multi-factor logistic regression results

| **Covariates** | OR | (95% CI) | P |
| --- | --- | --- | --- |
|  |  |  |  |
| Coronary artery disease | 2.81 | (1.71-4.61） | ＜0.001 |
| Diabetes mellitus | 1.26 | (0.76-2.07) | 0.369 |
| Hypertension | 2 | (1.30-3.06) | 0.002 |
| BMI≥28 | 0.71 | (0.36-1.41) | 0.324 |
| Cerebrovascular disease | 0.71 | (0.37-1.37) | 0.307 |

Supplementary Table 2 MINS related risk factors model

| Subgroup | Blood type | OR (95% CI) | P |
| --- | --- | --- | --- |
| Diabetes mellitus | Non-B |  |  |
|  | B | 1.08 (0.78-1.48） | 0.652 |
| Coronary heart disease | Non-B |  |  |
|  | B | 1.06 (0.73-1.54) | 0.747 |
| Hypertension | Non-B |  |  |
|  | B | 1.00 (0.78-1.29) | 0.977 |
